# Heat Treatment of Metallic Materials in Modern Industry

**DOI:** 10.3390/ma15238337

**Published:** 2022-11-23

**Authors:** Peter Jurči, Pavel Novák

**Affiliations:** 1 Faculty of Materials Science and Technology in Trnava, Slovak University of Technology, 91724 Trnava, Slovakia; 2 Department of Metals and Corrosion Engineering, University of Chemistry and Technology, Technická 6, 166 28 Prague, Czech Republic

The Heat Treatment of Metallic Materials in Modern Industry is a Special Issue of the journal *Materials*, which aims to publish original full-length articles and review papers on basic and applied research centered around the given topic, and thereby make the understanding of the metallurgical background of the contemporary state of heat treatment techniques used in the industrial branches in the 21st century.

Metals are still the most widely used materials in various branches of modern industry. In automotive industries, for instance, metallic materials make of around 75% of total weight of the middle-class vehicles such as Ford Mondeo, Audi A6, or Škoda Superb 3rd generation. For the proper functionality of components made of metallic materials, they must be subjected to different heat, thermo-chemical, or surface treatments. For these purposes, various types of equipment such as industrial furnaces, inductors, laser generators, electron beam, physical vapor deposition devices, 3D printers, and others have been used. Thermal- or thermo-chemical treatments evoke changes in bulk- or superficial microstructures of metals and thereby modify their properties. Changes in both the microstructures and properties of metallic materials should be carefully controlled. Different techniques and devices (such as light, electron, or confocal microscopes; hardness testers; and machines for the testing of wear and mechanical properties) are utilized in order to evaluate these alterations.

The papers which explore the research and development works on bulk heat and cryogenic treatment of steel, aluminium, titanium, magnesium, or copper alloys are particularly welcome. However, thermo-chemical treatments such as low-pressure carburizing, nitriding (including plasma processes), boronizing, and complementary techniques are also explored within this Special Issue. Finally, the papers on laser- or electron-beam surface treatments as well as the physical vapor deposition of thin films are of interest to the Editors of this Special Issue.

## Data Availability

Not applicable.

